# Rebamipide Enhances Pathogen Defense and Mitigates Inflammation in a Particulate Matter-Induced Ocular Surface Inflammation Rat Model

**DOI:** 10.3390/ijms26083922

**Published:** 2025-04-21

**Authors:** Basanta Bhujel, Se-Heon Oh, Woojune Hur, Seorin Lee, Hun Lee, Ho-Seok Chung, Jae Yong Kim

**Affiliations:** 1 Department of Ophthalmology, University of Ulsan College of Medicine, Asan Medical Center, Seoul 05505, Republic of Korea; basantabhujel86@gmail.com (B.B.); sh.oh@amc.seoul.kr (S.-H.O.); dnwnsgj@amc.seoul.kr (W.H.); ra02582@amc.seoul.kr (S.L.); yhun777@amc.seoul.kr (H.L.); 2 Department of Medical Science, University of Ulsan Graduate School, Seoul 05505, Republic of Korea

**Keywords:** particulate matter, rebamipide, hylauranic acid, ocular surface inflammation

## Abstract

Particulate matter (PM) exposure is known to induce significant ocular surface inflammation, necessitating effective therapeutic interventions. This study compared the efficacy of 2% rebamipide (REB) with 0.1% hyaluronic acid (HA) eye drops in investigating the anti-inflammatory and pathogen-clearance effects in a PM-induced ocular surface inflammation model using Sprague–Dawley (SD) rats. Parameters including clinical signs, histological changes, mucin secretions, inflammatory cytokines, mast cell degranulation, dysregulated cell proliferation, and cellular apoptosis were evaluated. 2% REB alleviated ocular surface inflammation by downregulating the nuclear factor kappa-light-chain-enhancer of activated B cells (NF-κB) inflammatory pathway and upregulating epidermal growth factor receptor (EGFR) signaling, thereby enhancing mucin secretion and promoting pathogen clearance. Histopathological analysis, western blot, and immunohistochemical staining revealed a marked reduction in inflammatory markers including MMP-9, IL-1β, TNF-α, IL-17, and CD-4, decreased mast cell degranulation, increased goblet cell density, and enhanced expression of mucins, including MUC5AC and MUC16, in the 2% REB-treated group compared to the 0.1% HA-treated and PM-exposed groups. Moreover, 2% REB demonstrated decreased apoptosis (TUNEL) and reduced uncontrolled cell proliferation (Ki67), indicating improved cellular integrity. In conclusion, 2% REB is a promising treatment option for PM-induced ocular surface inflammation in a rat model compared with 0.1% HA, offering the benefits of reducing inflammation, clearing pathogens, and protecting overall ocular health.

## 1. Introduction

Long-term exposure to air pollutants threatens human health and overall quality of life. Air pollution is composed of various constituents, such as particulate matter (PM), ozone (O_3_), sulfur dioxide (SO_2_), and nitrogen dioxide (NO_2_). However, PM has been noted as the most harmful of these pollutants [1]. PM is a heterogeneous mixture of airborne pollutants comprising both solid particles and liquid droplets suspended in the atmosphere. These particles vary significantly in size, chemical composition, and origin. Based on particle size, PM is commonly classified into PM_2.5_ (particles ≤ 2.5 μm) and PM_10_ (particles ≤ 10 μm), collectively referred to as fine PM. Exposure to PM can arise from combustion processes, including vehicle emissions, diesel exhaust, biomass burning, and various industrial activities. It can also stem from natural phenomena like wildfires and volcanic eruptions, along with human activities such as cigarette smoke and indoor fuel burning for cooking and heating [2]. Numerous epidemiological investigations have confirmed a robust association between airborne PM exposure and increased hospitalization and mortality rates, particularly affecting cardiovascular and respiratory health [3,4]. Despite these extensive studies, research on the effects of PM specifically on ocular health remains limited, highlighting a gap in current understanding regarding the eye’s susceptibility to environmental pollutants [5].

Ocular surface inflammation is a key pathological response that affects the cornea, conjunctiva, and tear film. It involves the activation of immune cells, promotes the release of proinflammatory cytokines, and compromises the integrity of the ocular surface. This inflammatory process can lead to symptoms like redness, discomfort, dryness, and visual disturbances [6]. Exposure to PM is a major cause of ocular surface inflammation, exacerbating these symptoms and compromising ocular health. Numerous rodent studies have demonstrated that topical PM administration in the eye induces inflammation, leading to symptoms like clinical dry eye disease (DED) or conjunctivitis [1,5,7,8,9,10]. Similarly, studies have demonstrated that prolonged exposure to PM can result in elevated levels of tear cytokines [11]. An epidemiological study manifested that exposure to air pollution decreased tear film stability and affected tear osmolarity [2]. Cui et al. reported that PM could hinder corneal epithelial wound healing by impairing cell migration [12]. Both in vitro and in vivo studies have demonstrated that PM triggers inflammation in corneal cells. The overexpression of inflammatory cytokines like interleukin (IL)-1α, IL-1β, IL-6, tumor necrosis factor (TNF)-α, and C-X-C motif chemokine ligand (CXCL) 2, alongside the activation of caspase-1, highlights this inflammatory response [8,13,14,15,16,17]. Additionally, the upregulation of high mobility group box (HMGB)-1, a proinflammatory molecule, and the activation of nuclear factor kappa-light-chain-enhancer of activated B cells (NF-κB), a key transcription factor in inflammation, were also observed [1,11]. Consistent with the biochemical studies, histological studies revealed that PM triggered both inflammatory and an allergic reaction in the cornea, including corneal limbus. This was characterized by increased neutrophil infiltration and alterations in conjunctival goblet cells [10,14,18]. A clinical study confirmed that PM provokes inflammation and promotes ocular surface neovascularization, as shown by the increased levels of IL-6, IL-8, and vascular endothelial growth factor (VEGF) [19,20]. An in vivo study showed that long-term PM exposure resulted in heightened inflammation of the conjunctiva, as indicated by a more significant increase in IL-6 levels compared to short-term exposure [21].

The current management for ocular surface inflammation comprises tear supplementation, tear stimulation, anti-inflammatory treatments, and environmental strategies to mitigate symptoms and restore overall ocular health. Presently, the foremost therapy for ocular surface inflammation is artificial tears, with anti-inflammatory therapies and punctal occlusion as secondary and tertiary treatment options. The primary aim of topical therapy with lubricating eye drops is to manage disease progression and activity, alleviate symptoms of ocular inflammation, and contribute to preventing or delaying ocular health consequences. Recently, rebamipide (REB) has been recognized for its potential in this context due to its anti-inflammatory properties and ability to enhance mucin production [22,23].

REB was introduced in Japan in 2012 under the brand Mucosta^®^ ophthalmic suspension (unit dose 2%), initially applied to treat DED and corneal damage [24]. Originally used to treat gastritis and gastric ulcers, REB is recognized for its anti-inflammatory, antioxidant, and promotion of gastric epithelial mucin effects [25,26,27,28]. Its application expanded to include treatment for ocular surface inflammation following its discovery of increasing mucin on the ocular surface. Studies found that REB increased mucin-like substances in the rabbit cornea and conjunctiva treated with N-acetylcysteine [28]. Furthermore, studies have shown that REB exhibits anti-inflammatory effects on ocular autoimmune lesions observed in a mouse model of Sjogren’s syndrome [29]. In addition, it also increases the number of goblet cells and reduces corneal inflammation [30,31,32]. Clinical research has demonstrated its effectiveness in alleviating symptoms and signs of DED [33,34,35,36]. In both clinical and pre-clinical settings, REB has been shown to increase the expression of mucin (MUC)-1, 4, 16, and 5AC genes by activating the epidermal growth factor receptor (EGFR), leading to increased production of mucin-like glycoproteins and goblet cells, along with a reduction in the inflammatory cytokines [37,38,39,40]. Previous outcomes manifested that topically applied REB significantly alleviated inflammatory symptoms, reduced corneal fluorescein staining, increased tear production, and improved tear film break-up time (TBUT) in DED patients [33,41,42,43]. Notably, studies have already proven that REB’s ability to modulate epithelial cell function, improve tear film integrity, and alleviate inflammation without any known major side effects makes it an ideal first-line therapy for treating severe dry eye and other ocular surface disorders [44].

Although previous studies have explored the beneficial impact of REB eyedrops on the ocular surface epithelium, tear function, and conjunctiva, its potential role in reducing inflammatory activity through pathogen clearance in PM-induced ocular surface inflammation has not yet been examined [38,39,41,45,46]. Thus, this study was designed to compare the therapeutic effects of 2% REB eye drops with 0.1% hyaluronic acid (HA) eye drops in ocular surface inflammation induced by PM exposure in Sprague–Dawley (SD) rats. In our study, 0.1% HA was administered due to its well-documented therapeutic effect on DED in Japan, as reported by a plethora of studies [34,47,48,49]. Furthermore, we compared the pathogen-clearance efficacy of 2% REB and 0.1% HA focusing on their abilities to upregulate EGFR signaling and downregulate NF-κB inflammatory pathways, thereby potentially mitigating ocular surface inflammation more effectively in a rat PM-induced ocular surface inflammation model.

## 2. Results

### 2.1. Effect of 2% REB on Ocular Surface Lesion

**Figure 1:**
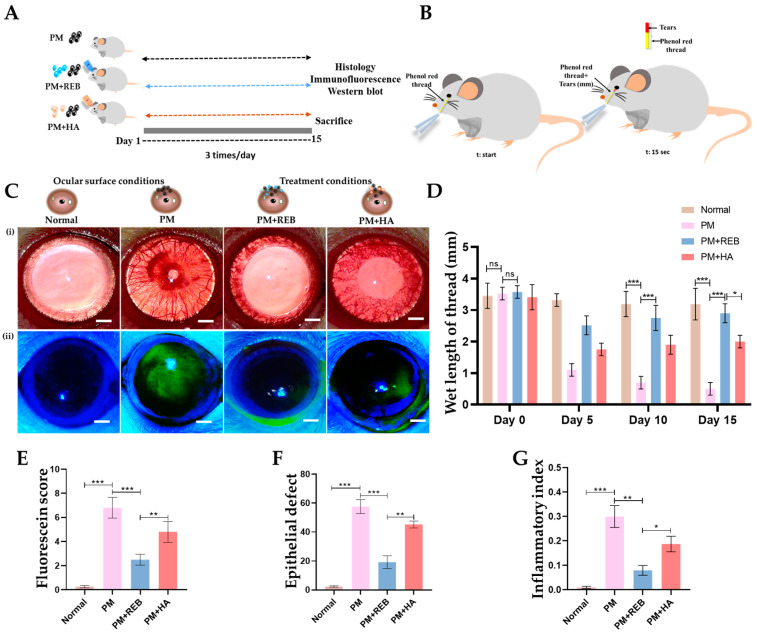
Representation of the topical application of 2% REB in PM-induced rat model of ocular surface inflammation. (**A**) Schematic demonstration of in vivo experimental plan. (**B**) Diagrammatic representation of the measurement of tearing rate by phenol red thread in millimeters (mm), with the thread placed at the time (t-start) and allowed to remain on the ocular surface for 15 s (t:15 s) before assessing the tear rate. (**C**) (**i**) Slit lamp examination of the ocular surface in the normal, PM-exposed, PM+REB, and PM+HA-treated groups (Scale bar: 2 mm). (**ii**) Fluorescein staining of the ocular surface in the normal, PM-exposed, PM+REB, and PM+HA-treated groups (Scale bar: 2 mm). (**D**) Tear-secretion rate (measured in mm wetted within 15 s) in the normal, PM-exposed, PM+REB, and PM+HA-treated groups in 0, 5, 10, and 15 days. (**E**) Changes in corneal fluorescein staining scores in the normal, PM-exposed, PM+REB, and PM+HA-treated groups. (**F**) Changes in epithelial defect of the ocular surface in the normal, PM-exposed, PM+REB, and PM+HA-treated groups. (**G**) Changes in inflammatory index in the normal, PM-exposed, PM+REB, and PM+HA-treated groups. In (**D**–**G**), the data are expressed as mean ± SEM (*n* = 10). One-way ANOVA was followed by the Tukey test. * *p* < 0.05, ** *p* < 0.01, *** *p* < 0.001 indicate statistically significant differences; ns, not significant.

The effect of 2% REB on ocular lesions was evaluated using fluorescein-stained score and epithelial defect area in the normal, PM-exposed, PM+REB, and PM+HA-treated groups in our PM-induced ocular surface inflammation rat model. The results showed a significant difference between the treatment groups (Figure 1C(ii)).

In terms of fluorescein score, the PM-exposed and PM+HA-treated groups exhibited significantly higher scores, indicating extensive ocular surface damage and epithelial disruption. The pronounced staining scores in these groups reflect a high level of inflammation and tissue damage. Conversely, the PM+REB-treated group demonstrated a markedly lower fluorescein staining score, suggesting that REB effectively reduced ocular surface damage and preserved epithelial integrity. The normal group showed no changes in fluorescein staining score, maintaining baseline levels and confirming the specificity of the induced inflammation and treatments (Figure 1E). Similar to our findings, the previous preclinical and clinical trials utilizing REB showed significantly greater improvement in the corneal fluorescein staining scores [43,50].

Further, the fluorescein-stained area was measured in our experimental groups. Our results revealed that the PM-exposed and PM+HA-treated groups showed a significant increase in the fluorescein-stained area, indicating widespread epithelial damage with extensive inflammation and tissue disruption. In contrast, the PM+REB-treated group had a significantly smaller fluorescein-stained area, indicating less epithelial damage and a protective effect of topical REB. The normal group showed no changes in the fluorescein-stained area, reinforcing that the observed effects were due to the induced inflammation and treatments (Figure 1F).

### 2.2. Effect of 2% REB on Tear Production

Next, we performed the phenol red thread test to evaluate the tear secretions and clearance effect in the normal, PM-exposed, PM+REB, and PM+HA-treated groups in our rat PM-induced ocular surface inflammation model. Our data revealed that the PM-exposed group showed a remarkably reduced tear secretion, followed by the PM+HA-treated group, indicating severe ocular damage, compromised tear film stability, and high inflammatory response. Interestingly, the PM+REB-treated group showed a marked improvement in tear secretions, suggesting a reduction in inflammation. The normal group showed a greater level of tear secretions compared to others (Figure 1B,D). These findings align with prior studies demonstrating the beneficial effects of 2% REB on tear production and stability [50].

### 2.3. Effect of 2% REB on Inflammatory Index

The inflammatory index was assessed in the normal, PM-exposed, PM+REB, and PM+HA-treated groups in our rat PM-induced ocular inflammation model. The results revealed that hyperemia and edema developed in the PM-exposed group, with similar but less pronounced features observed in the PM+HA-treated group. In contrast, the normal group showed no such inflammatory signs. Notably, the PM+REB-treated group exhibited a significant reduction in these inflammatory features (Figure 1C(i),G).

### 2.4. Effect of 2% REB on Mast Cell Degrnaulation in Conjunctiva

Next, the degranulated mast cells in the conjunctival tissue were investigated with toluidine blue staining in our rat PM-induced ocular surface inflammation model. Our previous study highlighted the significant role of mast cell activation in triggering the inflammation in PM-induced ocular surface inflammation [10]. Studies indicate that cytokines such as IL-1β and TNF-α, released by mast cells in the conjunctiva, play an essential role in initiating inflammatory reactions within the ocular surface [51].

**Figure 2:**
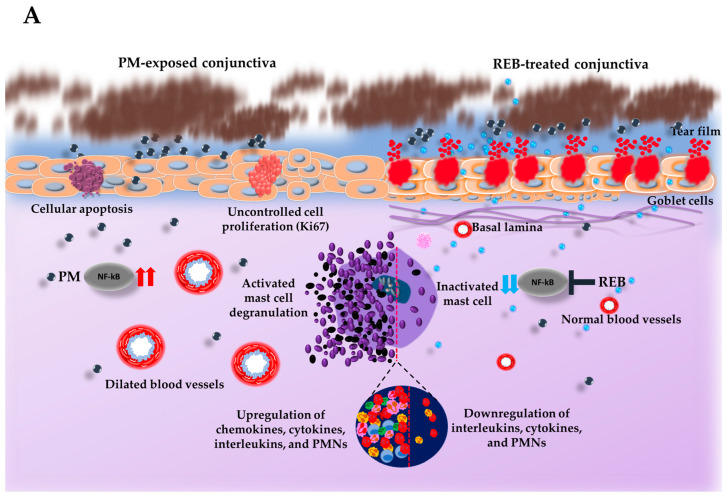

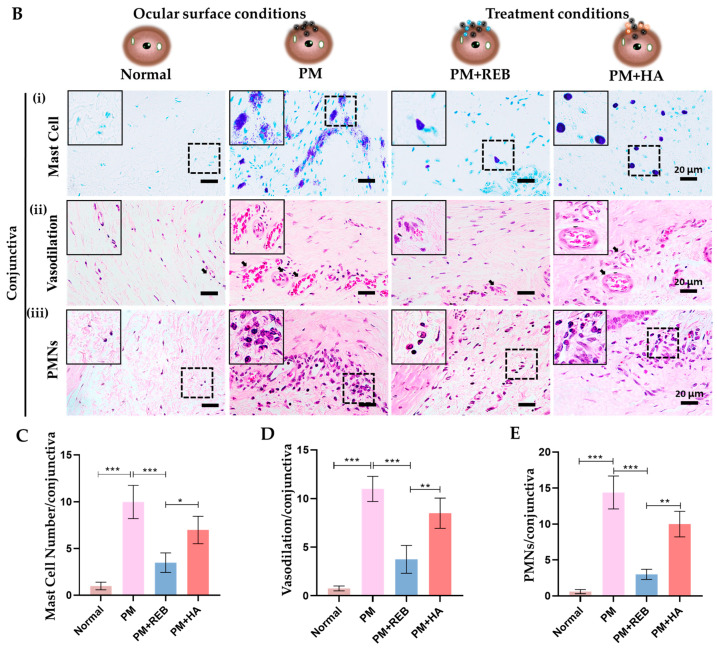
Topical 2% REB reduces mast cell degranulation, vasodilation, and PMNs in a PM-induced ocular surface inflammation rat model. (**A**) Schematic illustration of the impact of PM exposure and REB on the rat ocular surface. PM triggers mast cell degranulation and increases inflammatory cytokines by modulating NF-κB signaling, vasodilation, PMNs, cellular apoptosis, and uncontrolled cell proliferation, while 2% REB treatment reverses these effects, thereby promoting overall ocular health. (**B**) (**i**) Toluidine blue staining to evaluate degranulated mast cells in the normal, PM-exposed, PM+REB, and PM+ HA-treated groups. (**ii**) H&E staining to demonstrate the conjunctival vascular dilation in the normal, PM-exposed, PM+REB, and PM+HA-treated groups. (**iii**) H&E to manifest the conjunctival PMNs in the normal, PM-exposed, PM+REB, and PM+HA-treated groups. (**C**) Changes in the mast cell degranulation numbers, (**D**) vascular caliber, and (**E**) PMN cell infiltration in the conjunctiva. In (**B**) (**i**)–(**iii**) black rectangles in the left corner indicate the degranulated mast cells, dilated blood vessels, and PMNs in higher magnification, respectively. In (**C**–**E**), the data are expressed as mean ± SEM (*n* = 6). One-way ANOVA was followed by the Tukey test. * *p* < 0.05, ** *p* < 0.01, *** *p* < 0.001 indicate statistically significant differences.

From our findings, the normal group exhibited no degranulated mast cells. However, a marked increase in degranulated mast cells was found in the PM-exposed and PM+HA-treated groups. This heightened degranulation in the PM-exposed and PM+HA-treated groups underscores the severity of inflammation induced by PM exposure. Interestingly, very few degranulated mast cells were manifested in the PM+REB-treated group, suggesting its potential anti-inflammatory effects (Figure 2B(i),C).

### 2.5. Effect of 2% REB on Hemagiectasus in Conjunctiva

Similarly, H&E staining was carried out to reveal the hemagiectasis in the conjunctival tissue in our rat PM-induced ocular surface inflammation model. In our study, the PM-exposed group exhibited a considerable number of dilated blood vessels followed by the PM+HA-treated group. However, a noticeable reduction in dilated blood vessels was manifested in the PM+REB-treated group, whereas no vasodilation was seen in the normal group (Figure 2B(ii),D).

### 2.6. Effect of 2% REB on PMN Cell Infiltration in Conjunctiva

To demonstrate PMN cell infiltration in conjunctival tissue, H&E staining was conducted in our rat model of PM-induced ocular surface inflammation. PMN cells represent the pivotal cells in the ocular surface inflammations, playing a key role in delaying hypersensitivity responses mediated by T-cells in the conjunctiva [52].

From our finding, the PMN cell infiltration was notably more pronounced in the PM-exposed group. This was followed by the PM+HA-treated group, indicating a robust inflammatory response. In contrast, the PM+REB-treated group exhibited a substantial reduction in PMN levels, suggesting that REB effectively attenuated inflammation. The normal group maintained baseline PMN levels throughout the study (Figure 2B(iii),E).

### 2.7. Effect of 2% REB on Goblet Cell Numbers in Conjunctiva

Periodic Acid–Schiff (PAS) staining was assessed to study the distribution of goblet cell numbers in the conjunctival tissue in our rat PM-induced ocular surface inflammation model. Mucins produced by goblet cells contribute to tear film stability, lubrication, and protection against pathogens and environmental irritants [53].

**Figure 3:**
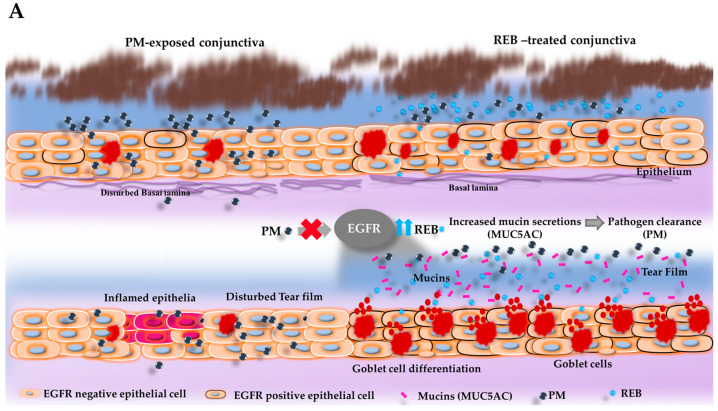

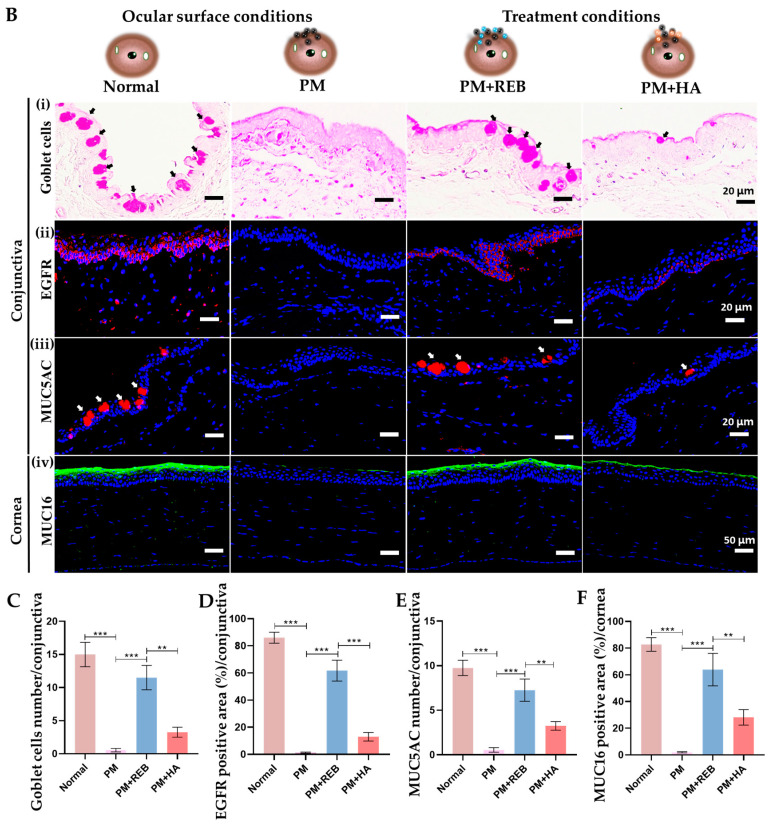
Topical 2% REB increases goblet cell numbers, EGFR expression, secreted-type mucin (MUC5AC) in the conjunctiva, and membrane-associated mucins (MUC16) in the cornea in the PM-induced ocular surface inflammation in a rat model. (**A**) Schematic illustration of the effects of PM and 2% REB on the goblet cells and subsequent mucin secretion (MUC5AC) in the rat conjunctiva. PM exposure in the ocular surface downregulates the EGFR expression in the epithelial cells, which leads to a decrease in the goblet cell differentiation and thus diminishes the goblet cell numbers. In contrast, topical 2% REB induces the EGFR expression, leading to goblet cell differentiation with an increment in goblet cell numbers. The rise in goblet cell count subsequently enhances the mucin secretion onto the ocular surface, suggesting its protective potential as a therapeutic agent in environments with high PM levels. (**B**) (**i**) PAS staining of rat conjunctiva was assessed to evaluate the distribution and the number of the goblet cell numbers in the normal, PM-exposed, PM+REB, and PM+HA-treated groups. (**ii**) Immunofluorescence staining of rat conjunctiva performed to manifest the EGFR expression in the normal, PM-exposed, PM+REB, and PM+HA-treated groups. (**iii**) Immunofluorescence staining of rat conjunctiva to show the MUC5AC expression level in the normal, PM-exposed, PM+REB, and PM+HA-treated groups. (**iv**) Immunofluorescence staining of rat cornea to show the MUC16 expression level in the normal, PM-exposed, PM+REB, and PM+HA-treated groups. (**C**) Changes in the goblet cell numbers, (**D**) EGFR expression, (**E**) MUC5AC expression in the conjunctiva, and (**F**) MUC16 expression in the cornea. In (**B**) (**i**) the black arrowheads denote the goblet cells in the conjunctiva. In (**B**) (**iii**) the white arrowheads indicate the expression of MUC5AC in the conjunctiva. In (**C**–**F**), data are expressed as mean ± SEM (*n* = 6). One-way ANOVA was followed by the Tukey test. ** *p* < 0.01, *** *p* < 0.001 indicate statistically significant differences.

Our findings revealed a substantial decrease in the goblet cell numbers in the both PM-exposed and PM+HA-treated groups when compared to the normal and PM+REB-treated groups. This decrease in goblet cell numbers in the PM-exposed and PM+HA-treated groups indicates impaired mucin production, likely due to inflammation induced by PM exposure. Conversely, treatment with 2% REB resulted in preserved goblet cell numbers, suggesting its potential to maintain mucin secretion and protect against ocular surface damage caused by PM exposure (Figure 3B(i),C).

### 2.8. Effect of 2% REB on EGFR Expression in Conjunctiva

Next, immunofluorescence staining was carried out to manifest the EGFR expression in conjunctival tissue in our rat PM-induced ocular surface inflammation model. Regulation of mucin production and ocular surface integrity is primarily mediated by EGFR. Upon activation, EGFR signaling accelerates mucin synthesis and secretion, essential for the lubrication and protection of the eye [54].

From our findings, higher EGFR expression was observed in the normal group. Interestingly, this expression was markedly downregulated in the PM-exposed group. Similarly, less expression of EGFR was noted in the PM+HA-treated group, while the PM+REB-treated group remarkably preserved the EGFR expression (Figure 3B(ii),D).

### 2.9. Effect of 2% REB in Secreted-Type Mucin (MUC5AC) in Conjunctiva and Membrane-Associated Mucins (MUC16) in the Cornea

Similarly, the immunofluorescence staining of MUC5AC was assessed in the conjunctival and MUC16 in the corneal tissue in our rat PM-induced ocular surface inflammation model. Mucins like MUC5AC and MUC16 are vital for overall ocular health, forming protective barriers and stabilizing the tear film [55]. They trap pathogens and debris, lubricate the ocular surface, and contribute to mechanical stress resistance [55]. They further contribute to the clearance mechanism of PM by aiding in the spread and distribution of the tears over the ocular surface, facilitating the effective removal of PM [55].

In our study, elevated levels of MUC5AC expression, primarily secreted by goblet cells to form a protective mucous barrier, were notably observed in both the normal and PM+REB-treated groups. Furthermore, MUC16 expression was prominently localized to the apical surface of the corneal epithelium in these groups. This elevated expression indicates effective mucin production and maintenance of the mucosal barrier in these groups. In contrast, the PM-exposed and PM+HA-treated groups exhibited significantly lower expression of MUC5AC and MUC16, suggesting a compromised ability to produce mucins essential for ocular surface protection, pathogen defense, and lubrication (Figure 3B(iii,iv),E,F).

### 2.10. Effect of 2% REB on Inflammatory Cytokines in Cornea and Conjunctiva

Furthermore, the immunofluorescence staining for MMP-9, TNF-α, IL-1 β, and IL-17 was performed in the corneal tissue in our rat PM-induced ocular surface inflammation model. Previous studies have confirmed the significant roles of these cytokines in ocular inflammation, with REB demonstrating efficacy in reducing these inflammation [39,49].

**Figure 4:**
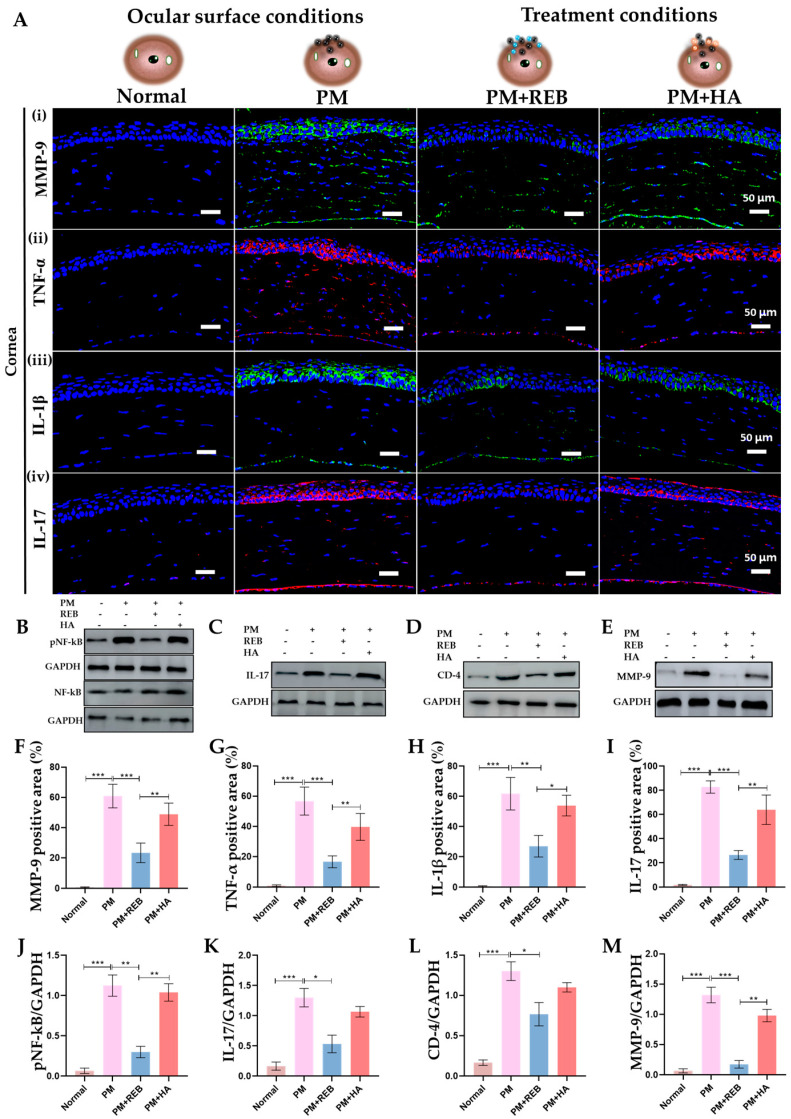
Topical 2% REB decreases the ocular inflammatory cytokines in PM-induced ocular surface inflammation in a rat model. (**A**) Immunofluorescence staining for (**i**) MMP-9, (**ii**) TNF-α, (**iii**) IL-1β, and (**iv**) IL-17 in the cornea of normal, PM-exposed, PM+REB, and PM+HA-treated groups. (**B**) Western blot analysis of pNF-κB and NF-κB, (**C**) IL-17, (**D**) CD-4, and (**E**) MMP-9 expression in the conjunctiva. (**F**) Changes in the expression of MMP-9, (**G**) TNF-α, (**H**) IL-1 β, and (**I**) IL-17 in the cornea. (**J**) Relative expression of pNF-κB, (**K**) IL-17, (**L**) CD-4, and (**M**) MMP-9 normalized to GAPDH. In (**F**–**M**), the data are expressed as mean ± SEM (*n* = 6, *n* = 4). One-way ANOVA was followed by the Tukey test. * *p* < 0.05, ** *p* < 0.01, *** *p* < 0.001 indicate statistically significant differences.

From our study, it was revealed that the levels of MMP-9, TNF-α, IL-1β, and IL-17 were markedly elevated in the PM-exposed and PM+HA-treated groups compared to the normal and PM+REB-treated groups. This heightened cytokine expression in the PM-exposed and PM+HA-treated groups indicates a strong inflammatory response induced by PM exposure. Conversely, treatment with 2% REB resulted in decreased levels of these inflammatory cytokines, suggesting its potential to mitigate PM-induced inflammation (Figure 4A(i–iv),F–I).

The western blot analysis of pNF-κB, IL-17, CD-4, and MMP-9 was carried out to evaluate the protein levels linked with inflammation in conjunctiva tissue in our ocular surface inflammation rat model. Our results showed a significant upregulation of protein levels in the PM-exposed group. A similar trend was detected in the PM+HA-treated group. Likewise, the protein expression of these inflammatory cytokines was dramatically diminished in the PM+REB-treated group resembling the normal group (Figure 4B–E,J–M). Like our findings, prior studies in the animal model revealed an upregulation of inflammatory cytokines on the ocular surface following PM exposure [5,10].

### 2.11. Effect of 2% REB on Cellular Apoptosis in Cornea and Conjunctiva

Additionally, cellular apoptosis in the corneal and conjunctival tissues was detected by TUNEL staining in a rat PM-induced ocular surface inflammation model. Prior research shows that PM causes cell apoptosis in the ocular tissues, leading to increased cell death and worsening eye inflammation and damage [7].

**Figure 5:**
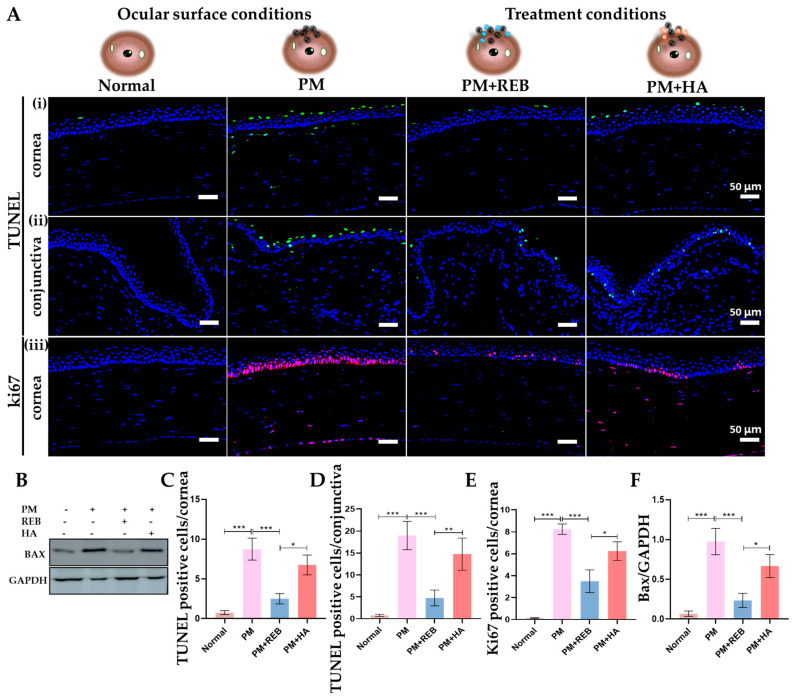
Topical 2% REB decreases cellular apoptosis and elevated cell proliferation in PM-induced ocular surface inflammation in a rat model. (**A**) (**i**,**ii**) TUNEL staining for cellular apoptosis in conjunctiva and cornea in normal, PM-exposed, PM+REB, and PM+HA-treated groups. (**iii**) Immunofluorescence staining for ki67 in the cornea in normal, PM-exposed, PM+REB, and PM+HA-treated groups. (**B**) Western blot analysis of Bax in normal, PM-exposed, PM+REB, and PM+HA-treated groups. (**C**,**D**) Changes in the TUNEL-positive cells in the cornea and the conjunctiva. (**E**) Changes in the ki67 positive cells percentage in the cornea. (**F**) Relative expression of Bax normalized to GAPDH. In (**C**–**F**), the data are expressed as mean ± SEM (*n* = 6, *n* = 4). One-way ANOVA was followed by the Tukey test. * *p* < 0.05, ** *p* < 0.01, *** *p* < 0.001 indicate statistically significant differences.

In our study, a significant rise in apoptotic cell death, marked by TUNEL-positive cells, was noted in the PM-exposed and PM+HA-treated groups compared to the normal and PM+REB-treated groups. This heightened apoptosis in the PM-exposed and PM+HA-treated groups reflects severe tissue damage induced by PM exposure. Conversely, treatment with 2% REB led to a reduction in apoptotic cells, suggesting its potential protective effect against PM-induced cellular damage and apoptosis (Figure 5A(i,ii),C,D).

The western blot analysis of Bax was performed in conjunctiva tissue to assess the protein levels related to cellular apoptosis in our rat PM-induced ocular surface inflammation model. Our findings demonstrated a marked rise in protein expression in the PM-exposed group with a similar pattern observed in the PM+HA-treated group. In contrast, protein expression was dramatically reduced in the PM+REB-treated group, closely resembling the levels observed in the normal group (Figure 5B,F).

### 2.12. Effect of 2% REB on Uncontrolled Cell Proliferation in Cornea

The immunofluorescence analysis for Ki67, a marker of abnormal cell proliferation, was performed on the corneal tissue of our rat PM-induced ocular surface inflammation model.

Our results demonstrated a notable increase in Ki67 cell expression in the PM-exposed group, followed by the PM+HA-treated group, within the cornea. In contrast, the PM+REB-treated group showed a dramatic reduction in Ki67-positive cells, closely resembling the normal group, where no proliferating cells were detected (Figure 5A(iii),E).

## 3. Discussion

To the best of our knowledge, this is the first study to investigate the anti-inflammatory effects of 2% REB in conjunction with its pathogen-clearance abilities in a rat PM-induced ocular surface inflammation model. Previous studies have explored the REB’s actions, revealing that it enhances mucus glycoprotein components, promotes migration of injured epithelial cells, upregulates the EGFR expression in unhealthy epithelium, and exhibits anti-inflammatory effects on both the gastric and ocular surface [56]. Based on these findings, this study explored the effects of commercially available 2% REB and 0.1% HA eye drops on tear secretion, ocular inflammatory cytokines, and both membrane-associated and secreted mucins in a rat model of PM-induced ocular surface inflammation, highlighting the ability of REB to enhance pathogen defense and mitigate inflammation. We believe that our study has thoroughly documented the findings on PM-induced ocular surface inflammation in a rat model, suggesting similar effects on the human ocular surface. The observed outcomes indicate potential improvements in subjective symptoms. Our results would be instrumental in establishing more potent and effective therapies for PM-induced ocular surface inflammation using 2% REB. The key outcomes of our study were as follows: the PM-exposed with 2% REB treatment (1) maintained the ocular surface integrity, (2) increased goblet cell density, (3) enhanced EGFR expression, (4) upregulated MUC5AC and MUC16 expression levels, (5) reduced mast cell degranulation, (6) decreased vasodilation, (7) lowered anti-inflammatory cytokines, (8) decreased cellular apoptosis and uncontrolled cell proliferation, and (9) diminished PMN cell infiltration.

Previous studies have already highlighted that 2% REB leads to significantly greater improvements in foreign body sensation and eye pain compared to 0.1% HA in the context of DED [34]. This finding aligns with the results observed in our study, where REB demonstrated similar efficacy in alleviating ocular surface inflammation. REB, known for its muco-protective properties, has been previously shown to enhance mucin secretion and stabilize tear film integrity in different ocular diseases [22,43]. This suggests that REB facilitates the clearance of PM, potentially through its influence on tear film dynamics and mucin production. Enhanced mucin secretion likely aids in the trapping and removal of PM from the ocular surface, thereby reducing the inflammatory burden [57]. Thus, the presumed action of topical 2% REB in the ocular surface was summarized in this present study. Our results are consistent with the existing research, highlighting EGFR as a central component in mucin production [54]. Topical REB application induces EGFR expression in the epithelial cells, which leads to the differentiation and increment of goblet cells in the conjunctiva [58]. This upregulation of EGFR also directly stimulates mucin secretion from these cells, as previously noted by numerous studies (Figure 3A) [59]. Specifically, the augmented population of conjunctival goblet cells subsequently elevates the production of secreted-type mucin (MUC5AC) and released into the tear fluid. The secreted-type mucin is critical for forming the protective viscous mucous layer on the ocular surface [38,44,57]. Similarly, a previous study demonstrated that following the administration of REB topically in a tear-deficient rabbit eye model, the increment of MUC5AC in the tear fluid was noticed markedly [58,59,60]. In addition, earlier research involving humans and animals showed that REB upregulates the positive goblet cell numbers and boosts mucin expression within the conjunctiva, thereby preventing ocular surface damage [29,44,46,57].

Notably, a study revealed that REB also stimulated the secretion of membrane-associated mucins by promoting mucin production in corneal epithelial cells [61]. The mucin, MUC16 serves as a protective barrier, preventing the corneal surface from exposure to pathogens, foreign particles, and possible trauma. It plays a vital role in boosting the corneal surface’s lubrication properties [44]. The secreted-type mucin aids in maintaining tear film stability, ensuring better protection and hydration through its interaction with the membrane-associated mucin [1]. These increased mucins (MUC5AC and MUC16) production and secretion, driven by REB treatment, enhance the mucous layer’s capacity to trap and clear PM, thus maintaining ocular surface homeostasis and moisture. A prior study confirmed the ability of REB to significantly upregulate MUC1 and MUC16 gene and protein expression in human corneal epithelial cell cultures [62].

Additionally, PM exposure has been shown to activate mast cells in the conjunctiva, leading to their degranulation and the subsequent secretion of inflammatory cytokines and mediators, including IL-4, TNF-α, IL-6, and IL-1β. These mediators promote to the inflammatory response and tissue damage observed in ocular surfaces exposed to PM [8,9,10]. The relationship between mast cells and PM is crucial, as mast cells amplify the inflammatory cascade, exacerbating the inflammation of the ocular surface [5].

In our study, we assessed the impact of 2% REB on NF-κB signaling in ocular surface inflammation induced by PM, revealing the downregulation of this pathway and emphasizing its significant role in inflammation suppression. Consistent with prior studies in mice models of ocular surface inflammation, our findings underscore that 2% REB treatment effectively reduces the inflammatory cascade, thereby mitigating the secretion of these inflammatory mediators. This highlights the potential of REB in breaking the cycle of inflammation by targeting mast cell-mediated inflammation, emphasizing its role in alleviating PM-induced ocular surface damage (Figure 2A) [50]. Numerous studies have already emphasized the critical role of this pathway in the progression of ocular diseases caused by PM exposure [1,5,10,63]. Similarly in a pre-clinical study in mice model of environmental dry eye stress (EDES) utilizing 2% REB and 0.1% HA, topical 2% REB showed decreased inflammatory cytokines, thereby showing a more favorable therapeutic effect in ocular inflammation than 0.1% HA [49]. Additionally, a study showed that REB treatment suppressed the activation of effector T cells. This resulted in a significant decrease in the release of TH1-type cytokines like IL-2 and IFN-γ, along with a reduction in NF-κB activity [29].

Supporting these findings, an in vitro study by Ueta et al. found that administering REB topically on the ocular surface in a dose-dependent manner could mitigate inflammation by reducing cytokine release from epithelial cells and PMN cell infiltration [64]. A prior investigation by Arakaki et al. demonstrated that topical application of REB effectively diminished inflammation in ocular autoimmune lesions within a murine Sjögren’s syndrome model, offering protective benefits to the ocular surface, aligning with the outcomes observed in our study [29].

The clinical study confirmed the effectiveness of topical REB in alleviating inflammation associated with ocular surface hypersensitivity conditions [65]. Further supporting this, a study in a murine model of experimental allergic conjunctivitis (EAC) demonstrated that topical REB significantly suppressed conjunctival inflammation and hypersensitivity responses on the ocular surface, highlighting its potential in treating inflammation in immune-mediated ocular conditions [22].

Besides, our study revealed that the corneal fluorescein staining, and inflammatory index were significantly reduced following 2% REB treatment. In contrast, these features were higher in the PM-exposed group treated with 0.1% HA, indicating that 2% REB significantly contributes to maintaining the tear film’s stability and structural integrity. The corneal and conjunctival epithelia are fundamental components of the ocular surface, acting as crucial defenses against pathogen invasion [66]. Damage to the corneal and conjunctival epithelia is one of the hallmarks of ocular surface inflammation progression [67,68]. Our results align with those of a prior clinical study that revealed the short-term benefits of 2% REB eye drops in improving corneal staining scores. This study also showed that topical application of REB ophthalmic solution for 2 weeks significantly improved corneal and conjunctival epithelial damage and relieved ocular symptoms in patients with DED. Further, compared to 0.1% HA eye drops, 2% REB treatment showed superior efficacy without causing serious side effects [34]. In another clinical study in 28 patients, comparing 2% REB and 0.1% HA in ocular surface diseases, 2% REB showed an effective outcome, thereby increasing tear production, fluorescein corneal staining score, improvement in conjunctival hyperemia, and corneal epithelial damage, consistent with our findings [69]. Another clinical study manifested that 2% REB effectively improved the conjunctival staining score after 4 weeks of treatment in comparison to 0.1% HA in patients with DED, thereby making it a safer and more effective treatment option than HA [70]. Eom et al. further demonstrated in a Phase 3 study that topical REB administration significantly improved corneal erosion healing, preserving corneal integrity [43]. These clinical results further support REB as a reliable and potent therapeutic option for ocular health conditions.

Likewise, 2% REB plays a significant role in mitigating cellular apoptosis in PM-induced ocular surface inflammation. This is crucial as PM exposure often triggers oxidative stress, leading to cellular apoptosis and subsequent inflammation. Studies have demonstrated that 2% REB exerts anti-inflammatory effects by downregulating pro-apoptotic pathways, thereby reducing the rate of cell death in ocular tissues in DED [44]. Additionally, 2% REB reduces the levels of Ki67, a protein linked to cell proliferation. Elevated levels of Ki67 are indicative of increased inflammatory cell activity, which exacerbates ocular surface inflammation. By reducing Ki67 expression, 2% REB effectively curtails the proliferation of inflammatory cells, thereby alleviating ocular surface inflammation. The dual action of REB in inhibiting apoptosis and uncontrolled proliferation underscores its therapeutic potential in managing PM-induced ocular surface inflammation, offering a protective mechanism against the adverse effects of environmental pollutants on the eyes.

Despite this, several modifications can be made to enhance the current model of ocular surface inflammation induced by PM exposure in rats. The rat model may not perfectly mimic human ocular physiology and responses to PM, REB, and HA, limiting the direct applicability of findings to human patients. Additionally, the specific dosages and methods of administration of PM, REB, and HA used in rats might not be applicable to humans, due to variations in pharmacokinetics and pharmacodynamics. The concentration and composition of PM in the study may not accurately reflect the varied and complex nature of PM exposure in real-world human environments. The duration of PM exposure and treatment in the rat model may not fully capture the chronic nature of PM exposure in humans, thus affecting the long-term assessment of treatment efficacy and safety. Moreover, coal fly ash and diesel exhaust particles are common components of airborne PM, frequently used in studies to examine PM’s impact on biological functions. Therefore, additional research is necessary to evaluate how these distinct types of airborne particles affect the ocular surface compared to real PM, to enhance our understanding of PM’s impact on the ocular surface [55,71]. Biomarkers used to assess inflammation in rats might not be entirely representative of those in humans, leading to discrepancies in interpreting the anti-inflammatory efficacy of the treatments. Furthermore, while rats can be used to study physiological and biochemical responses, assessing subjective symptoms such as discomfort and visual disturbances, which are relevant to human ocular inflammation, is challenging. In addition, our study may not capture all potential side effects and long-term safety concerns of REB and HA when used in a clinical setting. Likewise, the exact molecular mechanism behind the therapeutic benefits of topical REB treatment remains incompletely understood, and further research is mandatory to establish an effective treatment for ocular surface inflammation [29].

Nevertheless, 2% REB has demonstrated superior efficacy compared to 0.1% HA in alleviating PM-induced ocular surface inflammation, underscoring the significance of our findings in advancing therapeutic strategies for ocular surface diseases. This study provides important insights into the inflammatory processes triggered by PM exposure and the potential of REB to mitigate these effects. Furthermore, these outcomes establish a foundation for future translational research focused on exploring the dual role of REB in both reducing inflammation and enhancing pathogen defense in individuals exposed to environmental PM. Such investigations could contribute to the development of more effective treatment modalities for managing ocular surface inflammation in populations at risk due to ambient PM exposure.

## 4. Materials and Methods

### 4.1. Animals and Experimental Design

A total of 40 female, 8-week-old SD rats were purchased from Orient Bio (Seongnam, Republic of Korea) The rats were acclimatized for at least 1 week before the experiment, maintained in a 12-h light/dark cycle, and given free access to autoclaved food and water. All experimental procedures were conducted in accordance with the guidelines of the Institutional Animal Care and Use Committee (IACUC) of Ulsan University (IACUC 2023-10-176), and principles of laboratory animal care.

The rats were randomly assigned into four experimental groups: (1) normal, (2) PM-exposed without treatment, (3) PM-exposed with 2% rebamipide (PM+REB), and (4) PM-exposed with 0.1% Hyaluronic Acid (PM+HA) (*n* = 10/each group). Except for the normal group, all PM-exposed groups (PM, PM+REB, and PM+HA) were subjected to 3 mg/mL of PM (SRM 2786; Sigma-Aldrich, Taufkirchen, Germany) three times daily for 15 days. The PM+REB group was treated with 2% REB (Reba-K, Samil Co. Ltd., Seoul, Korea) and the PM+HA group was treated with 0.1% HA, both administered three times daily for 15 days (Figure 1A).

All the rats were humanely euthanized on day 16 using CO_2_ asphyxiation, and their left whole eye was collected for analysis.

### 4.2. Inflammation Indexing

The ocular manifestations of inflammation were evaluated based on three different parameters utilizing a slit lamp microscope as previously described [72]. In brief, the inflammatory index was assessed by examining three major features: ciliary hyperemia, central corneal edema, and peripheral corneal edema. Each was scored from 0 to 3 according to severity, with higher scores indicating more pronounced inflammation [5,73].

### 4.3. Tear Secretion Measurements

Tear volume was determined using a Zone-Quick phenol red cotton thread, as described previously [74]. In unanesthetized animals, a cotton phenol red thread was applied in the lateral conjunctival fornix for 15 s. The thread changed from yellow to red as it absorbed tear fluid, and the tear distance was measured using a caliper (Monos, Seoul, Korea) [74]. Tear fluid volumes were measured on days 0, 5, 10, and 15 (Figure 1B).

### 4.4. Corneal Fluorescein Staining and Scoring

A 1% fluorescein solution (Sigma-Aldrich, Germany) was applied to the corneal surface of the rats. After 2 min, excess fluorescein was rinsed off using artificial tears (HAI Laboratories, Lexington, MA, USA) to prevent false positives. To analyze corneal staining, the eye was imaged in a dark room using a microscope equipped with cobalt blue illumination. Quantification of punctate staining was performed using ImageJ software (v1.62f).

Fluorescein staining was evaluated using a grading scale from 0 to 4, based on the intensity of ocular staining. Three independent observers performed semi-quantitative scoring in a blinded manner. The corneal fluorescein staining was classified according to the extent of the stained area: grade 0 for no staining, grade 1 for staining of up to one-eighth of the cornea, grade 2 for up to one-half of the cornea stained, and grade 4 for staining of more than half of the entire corneal surface [75,76].

### 4.5. Hematoxylin and Eosin (H&E) Staining

The entire left eye was removed for histological examination to assess PMN cell infiltration and vasodilation in the conjunctival tissue. The tissue samples were then embedded in paraffin and sectioned at 5 µm by a microtome (Leica, Wetzlar, Germany). These sections were deparaffinized, dehydrated, and stained with H&E solution (Abcam, Cambridge, UK). Following staining, the sections were cleared with xylene and mounted for microscopic observation.

### 4.6. Toluidine Blue Staining

The tissue sections of 5 µm thickness were prepared using a microtome (Leica, Wetzlar, Germany), and the prepared sections were subjected to a 1% toluidine blue (Daejeon, Republic of Korea) staining solution for 5–8 min. The stained sections were then washed, dehydrated, and mounted for further analysis. Upon microscopic examination, distinct purple or blue granules were observed, indicating mast cell degranulation.

### 4.7. Immunofluorescence Staining

Immunohistochemical analysis of the conjunctival and corneal tissues was performed after sacrificing the rats via a CO_2_ chamber. The harvested tissues were prepared for paraffin embedding, and 5 µm sections were made. For immunostaining, the sections were rehydrated and then incubated with primary antibodies at 4 °C against IL-17 (ab79056; Abcam, Cambridge, UK, 1:100), TNF-α (3707; Cell Signaling Technology, Danvers, MA, USA, 1:200), Ki67 (ab15580; Abcam, Cambridge, UK, 1:200), MMP-9 (ab73734; Abcam, Cambridge, UK, 1:200), MUC5AC (MA5-12178; Thermo Fisher Scientific, Waltham, MA, USA, 1:50), MUC16 (ab133419; Abcam, Cambridge, UK, 1:50), IL-1β (ab315084; Abcam, Cambridge, UK, 1:100), and EGFR (4267S; Cell Signaling Technology, Danvers, MA, USA, 1:100). After 24 h of incubation, sections were washed with PBS and again incubated with Alexa Fluor 488 (A11008; Invitrogen, Waltham, MA, USA, 1:400), Alexa Fluor 555 (A21424; Invitrogen, Waltham, MA, USA, 1:400), Alexa Fluor 568 (A10042; Invitrogen, Waltham, MA, USA, 1:400) and Alexa Fluor 488 (A11029; Invitrogen, Waltham, MA, USA, 1:400)-conjugated secondary antibodies in the dark at room temperature for 1 h. Nuclear staining was carried out using DAPI for 10 min and mounted. Images were visualize using a confocal microscope (Carl Zeiss, Jena, Germany). ImageJ (v1.62f) was employed for subsequent image analysis.

### 4.8. Periodic Acid-Schiff (PAS) Staining

The tissue sections of 5 µm thickness were prepared using a microtome (Leica, Wetzlar, Germany) and stained with 1% PAS solution (Sigma-Aldrich, Burlington, MA, USA), followed by Schiff’s Reagent (Sigma-Aldrich, Burlington, MA, USA) [10]. After counterstaining with Harris Hematoxylin, the slides were rinsed, dehydrated, cleared, and mounted for microscopic observation.

### 4.9. TUNEL Assay

The conjunctival and corneal tissues were embedded in paraffin, and a 5 µm section was made using a microtome (Leica, Wetzlar, Germany). The tissues were subjected to dewaxing and dehydration before performing TUNEL (Terminal deoxynucleotidyl transferase dUTP nick-end) labeling (684817910; Roche, Munich, Germany). Following nucleus staining, images were captured using a confocal microscope (Carl Zeiss, Jena, Germany), and the number of apoptotic cells were quantified.

### 4.10. Western Blotting

The conjunctival tissues were carefully excised after sacrificing rats in a CO_2_ chamber. The tissue samples were manually homogenized in a lysis buffer containing protease inhibitors. Proteins were separated by SDS-PAGE, followed by transfer onto PVDF membranes. Membranes were then incubated at 4 °C condition overnight with primary antibodies against pNF-κB (3033S; Cell Signaling Technology, Danvers, MA, USA, 1:1000), NF-κB (8242S; Cell Signaling Technology, Danvers, MA, USA, 1:1000), CD-4 (NBP1-19371; Novus Biologicals, Littleton, CO, USA, 1:1000), Bax (sc-20067; Santa Cruz Biotechnology, Dallas, TX, USA, 1:1000), IL-17 (ab79056; Abcam, Cambridge, UK, 1:1000), MMP-9 (ab73734; Abcam, Cambridge, UK, 1:1000) and GAPDH (2118S; Cell Signaling Technology, Danvers, MA, USA, 1:10,000). The membranes were then rinsed three times with TBST and incubated for 1 h at room temperature with a secondary antibody conjugated to HRP. Bands were detected using a chemiluminescence system (WBKLS0100; MilliporeSigma, St. Louis, MO, USA). ImageJ software (version 1.62f, https://imagej.nih.gov/ij/; accessed on 2 January 2025; developed by Wayne Rasband, National Institutes of Health, Bethesda, MD, USA) was used for the analysis of protein expression.

### 4.11. Statistical Analysis

The data are presented as mean ± standard error of the mean (SEM). Data were analyzed with GraphPad Prism 5.01 (GraphPad Software, Boston, MA, USA) and quantified using ImageJ (version 1.62f, https://imagej.nih.gov/ij/; accessed on 2 January 2025; developed by Wayne Rasband, National Institutes of Health, Bethesda, MD, USA). The statistical analysis between groups was evaluated using one-way ANOVA and the Tukey test. Bartlett’s test assessed the impact of multiple treatments in in vivo experiments, with *p* < 0.05 indicating significance.

## 5. Conclusions

This study provides compelling evidence that 2% REB enhances pathogen clearance and attenuates PM-induced ocular surface inflammation in a rat model. The REB effectively reduced inflammatory markers and improved the condition of the ocular surface. These promising findings underscore the high potential efficacy of 2% REB than 0.1% HA as a therapeutic intervention in ocular surface diseases exacerbated by environmental pollutants. Future research should focus on elucidating the detailed molecular mechanisms by which REB enhances pathogen clearance and further explore its efficacy in clinical settings involving human subjects exposed to PM pollutants. Comprehensive clinical trials and studies on diverse populations and environmental conditions are essential to fully establish the therapeutic potential of REB.

## Data Availability

All datasets used and/or analyzed during the current study are available from the corresponding author on reasonable request.

## Abbreviations

PM	Particulate Matter
REB	Rebamipide
HA	Hyaluronic Acid
TNF	Tumor Necrosis Factor
CXCL	C-X-C motif chemokine ligand
NF-kB	Nuclear Factor Kappa-Light-Chain-Enhancer of Activated B Cells
VEGF	Vascular Endothelial Growth Factor
DED	Dry Eye Disease
MUC	Mucin
EGFR	Epidermal Growth Factor Receptor
TBUT	Tear Film Break-Up Time
SD	Sprague-Dawley
IACUC	Institutional Animal Care and Use Committee
PAS	Periodic Acid–Schiff
EDES	Environmental Dry Eye Stress
PMNs	Polymorphonuclear Leukocytes
IL-4	Interleukin-4
Il-17	Interleukin-17
